# New records of water mites of the family Sperchontidae Thor, 1900 from China (Acari, Hydrachnidia), with descriptions  of two new species

**DOI:** 10.3897/zookeys.158.1970

**Published:** 2011-12-22

**Authors:** Xu Zhang, Dao-Chao Jin

**Affiliations:** 1 The Provincial Key Laboratory of Agricultural Pest Management in Mountainous Region, Institute of Entomology, Guizhou University; Guiyang 550025, China; 2 Anhui Key Laboratory of Plant Resources and Biology, Huaibei Normal University; Huaibei 235000, China

**Keywords:** Hydrachnidia, Sperchontidae, new species, new records, China

## Abstract

Five species of water mites of the family Sperchontidae Thor, 1900 are reported from China. Two of them are new to science, *Sperchon (Sperchon) orbipatella* **sp.n.** and *Sperchon (Sperchon) urumqiensis* **sp. n.**, and the other three are new to China, i.e., *Sperchon (Palpisperchon) nikkoensis* Imamura, 1976, *Sperchon (Sperchon) sounkyo* Imamura, 1954 and *Sperchonopsis (Sperchonopsella) whiteshellensis* Conroy, 1991. The first descriptions of the female of *Sperchon nikkoensis* and the male of *Sperchon sounkyo* are also given. The subgenus *Sperchonopsella* Conroy is new to the fauna of China.

## Introduction

Water mites of the family Sperchontidae Thor, 1900 are presently known from all biogeographic regions, except Antarctica, and over 200 species were reported (Cook 1974, Viets 1987, Di Sabatino et al. 2008). Up to now, 17 species were described from China: *Sperchon beijingensis* Zhang & Jin, 2010; *Sperchon brevipalpis* Jin, 1997; *Sperchon curvipalpis* Zhang & Jin, 2010; *Sperchon fluviatilis* Uchida, 1934; *Sperchon garhwalensis* Kumar, Kumar & Pesic, 2007; *Sperchon gracilipalpis* Lundblad, 1941; *Sperchon heteropoda* Zhang & Jin, 2010; *Sperchon huangshanensis* Zhang & Jin, 2010; *Sperchon lanigerus* Guo & Jin, 2011; *Sperchon mirabilis* Lundblad, 1941; *Sperchon oligospinis* Jin, 1997; *Sperchon placoderma* Lundblad, 1967; *Sperchon perspicuus* Zhang & Jin, 2011; *Sperchon plumifer* Thor, 1902; *Sperchon rostratus* Lundblad, 1968; *Sperchon turfanensis* Zhang &Jin, 2010 and *Sperchonopsis echphyma* Prasad & Cook, 1972 (Zhang and Jin 2010, Jin 1997; Zhang et al. 2010, Jin et al. 2010, Zhang et al. 2007, Zhang et al. 2011).

During checking our collection of water mites from China, five sperchonthid species were found. Two of them, *Sperchon (Sperchon) orbipatella* sp. n. and *Sperchon (Sperchon) urumqiensis* sp. n., are new to science. The other three taxa, i.e., *Sperchon (Sperchon) sounkyo* Imamura, 1954, *Sperchon (Palpisperchon) nikkoensis* Imamura, 1976 and *Sperchonopsis (Sperchonopsella) whiteshellensis* Conroy, 1991, are new records for China. The subgenus *Sperchonopsella* is new to the fauna of China.

## Material and methods

Specimens were collected by Dao-Chao Jin, Jian-Jun Guo, Zhen-Zao Tian, Ai-Ping Cui and Xu Zhang during 1997–2010 from China, and preserved in Koenike’s fluid and dissected as described elsewhere (e.g. Cook 1974). Terms follow Jin (1997). The following abbreviations are used:

A1, A2 = antennal glandularia 1 and 2; ACG = anterior coxal group (CxI + CxII); CxI–CxIV = coxae I–IV; D1–D4 = dorsoglandularia 1–4; E1–E4 = epimeroglandularia 1–4; L1–L4 = lateroglandularia l–4; O1, O2 = ocularia l and 2; PCG = posterior coxal group (CxIII + CxIV); P-I–P-V = palpal segments 1–5; V1–V4 = venteroglandularia 1–4; I-L-1–I-L-6 = the first leg segments 1–6; II-L-1–II-L-6 = the second leg segments 1–6; III-L-1–III-L-6 = the third leg segments 1–6; IV-L-1–IV-L-6 = the fourth leg segments 1–6.

All the type specimens are deposited in the Institute of Entomology, Guizhou University, China (GUGC).

All measurements are given in µm with the mean first, followed by the range in bracket.

## Systematics

**Family Sperchontidae Thor, 1900**

### Genus *Sperchon* Kramer, 1877

#### Subgenus *Palpisperchon* Lundblad, 1941

##### Sperchon (Palpisperchon) nikkoensis

Imamura, 1976

http://species-id.net/wiki/Sperchon_nikkoensis

**Figures 1–3.**
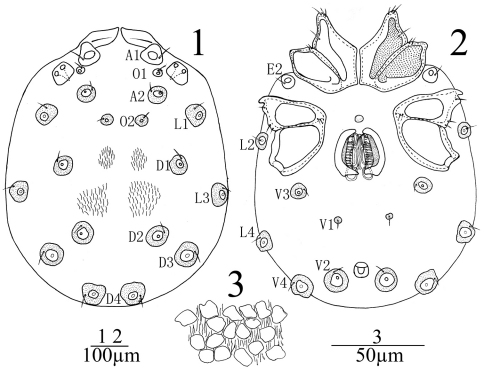
*Sperchon (Palpisperchon) nikkoensis*, Imamura, 1976, Male **1** idiosoma, dorsal view **2** idiosoma, ventral view **3** decorations of cuticle.

Figures 1 [Fig F2] 10 

###### Material examined.

2 females, Hainan Province, Bawangling National Nature Reserve, an unnamed stream (19°07'16"N, 109°04'58"E), 15 August 2005, coll. Xu Zhang; 1 male and 2 females, Guizhou Province, Leigongshan National Nature Reserve, an unnamed stream (26°21'06"N, 108°12'39"E), 3 October 2005, coll. Xu Zhang; 2 males and 2 females, Anhui Province, Jinzhai city, Shuanghe country, an unnamed stream (31°36'37"N, 115°41'30"E), 19 July 2010, coll. Xu Zhang; 1 female, Anhui Province, Anqing city, Mingtangshan scenic area, Hulu River (30°51'19"N, 116°06'06"E), 20 August 2010, coll. Xu Zhang.

###### Description.

**Male** (n = 3): Idiosoma flat, 730 (730-768) in length, 583 (583-616) in width, color yellow-brown. Cuticle soft and covered with small flat papillae and fine striations in various form and size (Fig. 3). Dorsum without chitinous plate, only two pair of muscular sigillae faintlyvisible. Each glandularia on dorsum and venter encircled by a plate and raised conically as nipples. The anterior dorsal area before A1 protruded dorsally forward and leaf-shaped with a vein-like line in dorsal view. Coxae in four groups, surface of coxae reticulated. ACG 210 (210-218) in length, close to each other but not fused, posterior apodeme indistinct. E2 laterally between ACG and PCG; PCG 213 (213–230) in length, widely separated. Glandularia absent from CxIII. Distance between anterior end of ACG and posterior end of PCG 438 (438-451). Genital field between CxIV of PCG with a small and rounded platelet in front. Genital valves not covering the genital acetabula, 143 (143-152) in length, 135 (135-140) in width. Three pairs of acetabula, the anterior two elliptic and the posterior more or less rounded. Cuticle with fine striations between the genital organ and coxal groups. V1 without accompanying glandularia but on small sclerites. Excretory pore surrounded by a sclerotized ring and close to the line of V2.

Infracapitulum with a short rostrum, length 292 (292-236). Chelicera total length 352 (352-387), basal segment length 283(283-308), claw length 69 (69-79), basal segment/claw length ratio 4.1 (3.9-4.1). Palp short and thick. Dorsal lengths of the palpal segments: P-I, 35 (35-37); P-II, 114 (114-121); P-III, 48 (48-53); P-IV, 89 (89-98); P-V, 52 (52-59). P-I stout and without seta. P-II thick with a long ventro-distal projection, bearing three setae, one of which nearly at the base of the projection and slightly longer than projection, the other two relatively short located approximately on the middle of projection. Nine seta on the dorsal and lateral side of the P-II. P-III shorter than P-II, with a long and thin ventrodistal seta and three short dorsal setae. P-IV with two greatly enlarged ventral peg-like setae, close to each other.

Dorsal lengths of leg I: I-L-1, 39 (39-48); I-L-2, 72 (72-81); I-L-3, 83 (83-97); I-L-4, 137 (137-151); I-L-5, 156 (156-171); I-L-6, 127 (127-141). Dorsal lengths of leg IV: IV-L-1, 88 (88-97); IV-L-2, 95 (95-110); IV-L-3, 129 (129-145); IV-L-4, 237 (237-255); IV-L-5, 220 (220-236); IV-L-6, 192 (192-208). Ambulacrum with two claws. Claws with well protruded claw-blade and two clawets, a long dorsal and a shorter ventral one (Fig. 8).

**Female** (n = 3): Similar to male except for the morphology of genital field and the size of idiosoma. Idiosoma 816 (792-857) in length, 688 (643-712) in width. ACG 310 (302-317) in length, PCG 319 (308-336) in length. Distance between anterior end of ACG and posterior end of PCG 606 (587-624). Genital field 234 (226-242) in length, 215 (208-229) in width. Pregenital sclerite crescent-shaped, and more developed than the postgenital sclerite. Infracapitulum length 301 (290-316). Chelicera total length 381 (364-409), basal segment length 308 (293-328), claw length 73 (71-80), basal segment/claw length ratio 4.2 (4.1-4.2). Dorsal lengths of the palpal segments: P-I, 41 (40-47); P-II, 123 (119-128); P-III, 57 (55-62); P-IV, 94 (89-99); P-V, 57 (55-64). Dorsal lengths of the first leg: I-L-1, 54 (52-62); I-L-2, 80 (78-92); I-L-3, 103 (99-116); I-L-4, 157 (149-176); I-L-5, 179 (168-194); I-L-6, 111 (106-120). Dorsal lengths of the fourth leg: IV-L-1, 72 (70-88); IV-L-2, 123 (117-138); IV-L-3, 149 (138-162); IV-L-4, 268 (257-285); IV-L-5, 266 (259-281); IV-L-6, 208 (200-217).

###### Remarks.

At present, only fivespecies of the subgenus *Palpisperchon* Lundblad, 1941 are known: *Sperchon crassipalpis* Marshall, 1933, *Sperchon distans* Scheffler, 1972, *Sperchon mirabilis* Lundblad, 1941, *Sperchon nikkoensis* Imamura, 1976 and *Sperchon skopetsi* Tuzovskij, 1982.

Due to the shape of cuticle, E4 absent from CxIII, P-II with a very long ventro-distal projection, and P-IV with two greatly enlarged ventral peg-like setae, the specimens from China show a general conformity with *Sperchon (Palpisperchon) nikkoensis*, a species previously known only from the female sex from Japan (Imamura 1976). However, the absence of pregenital sclerite and the presence of a round platelet in front of the genital field (as reported in the original illustration, see Imamura 1976), are typical characters of the male sex (Di Sabatino et al. 2010). Therefore, the specimen described by Imamura should be a male instead of female. This is the first description of the opposite sex to that in original description (Imamura 1976), and first record of this species from China.

**Figures 4–8. F2:**
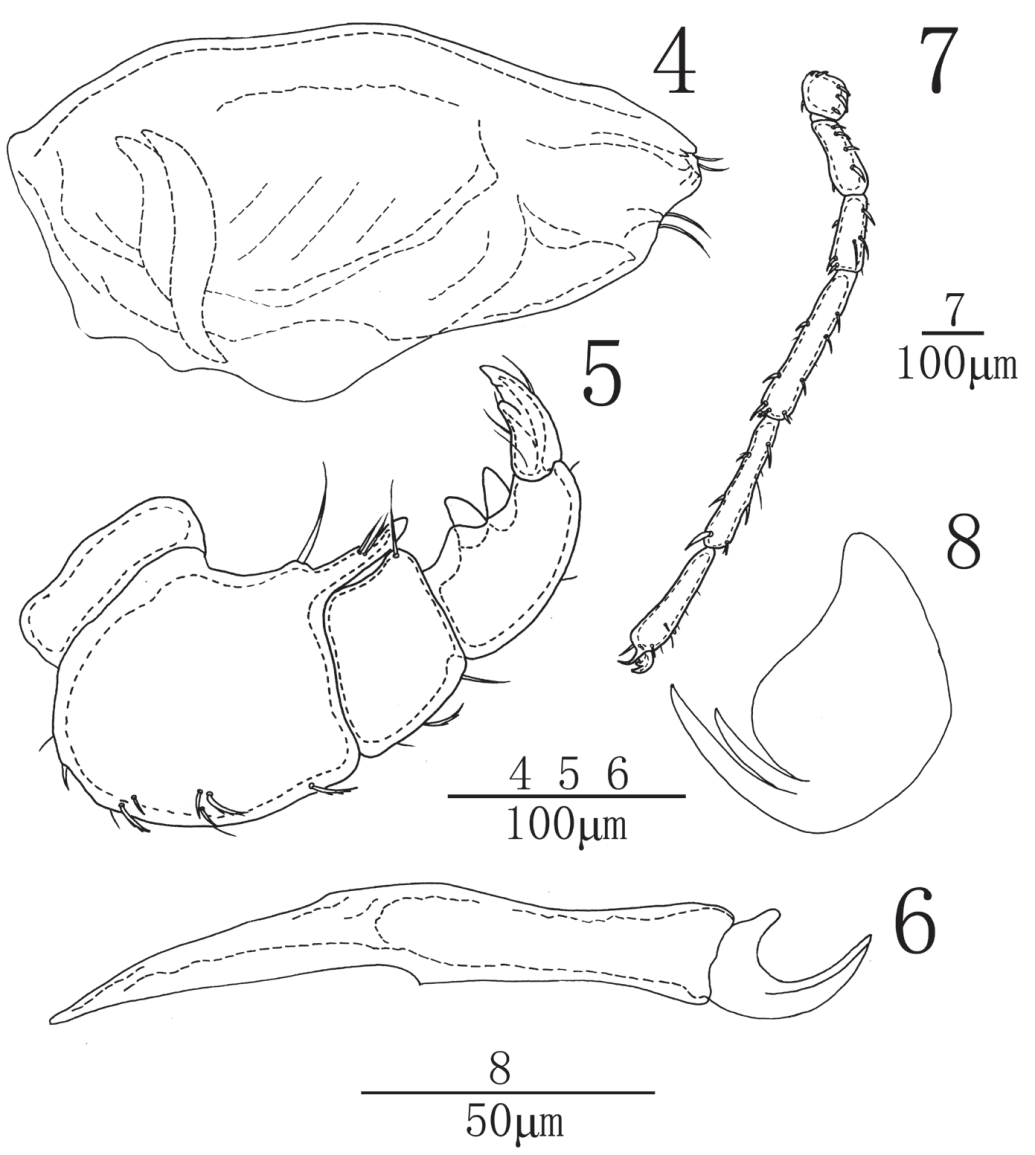
*Sperchon (Palpisperchon) nikkoensis*, Imamura, 1976, Male **4** infracapitulum **5** palp **6** chelicera **7** IV-L-1–6; **8** claw.

**Figures 9–10.**
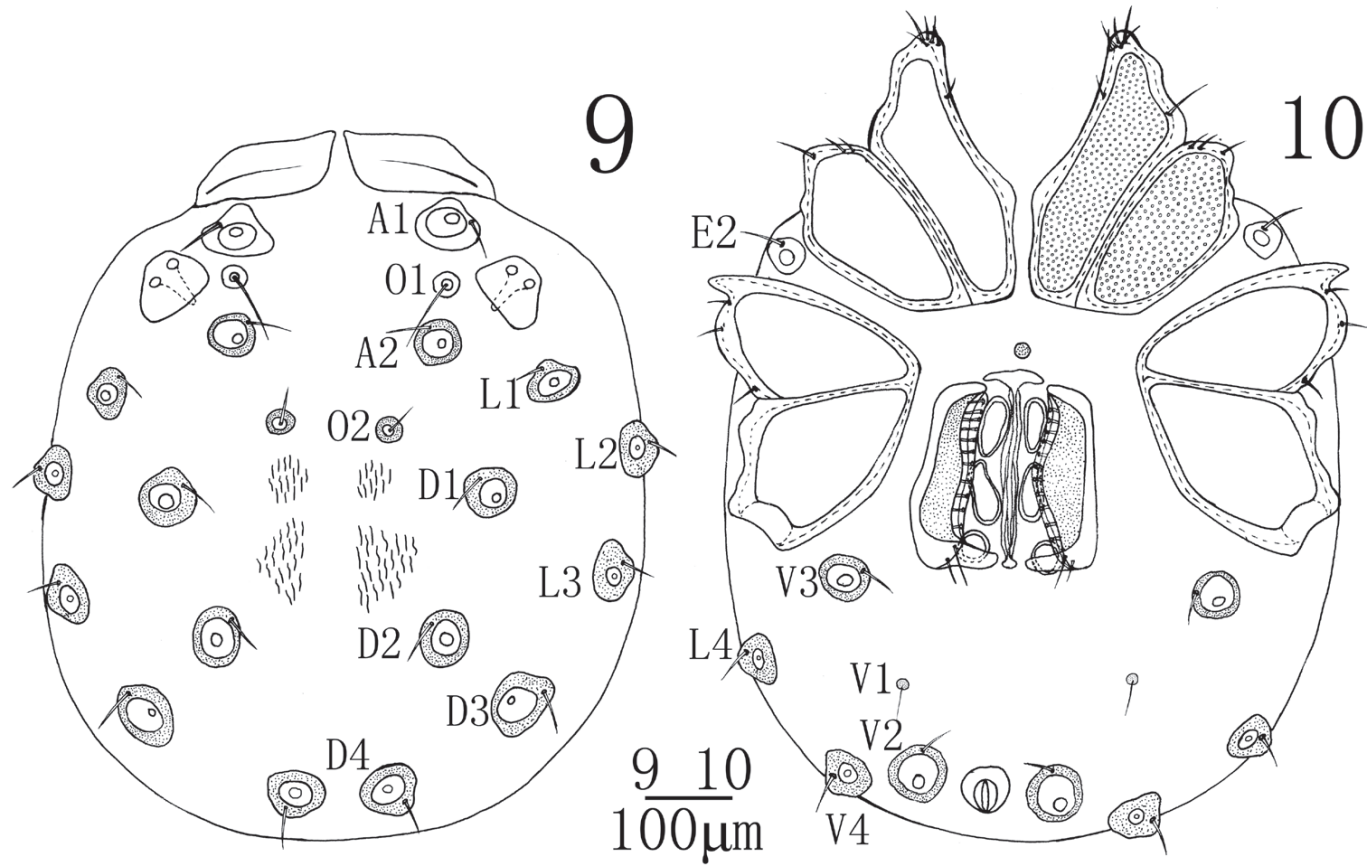
*Sperchon (Palpisperchon) nikkoensis*, Imamura, 1976, Female **9** idiosoma, dorsal view **10** idiosoma, ventral view.

###### Distribution.

China (present study); Japan (Imamura 1976).

#### Subgenus *Sperchon* Kramer, 1877

##### Sperchon (Sperchon) orbipatella sp. n.

urn:lsid:zoobank.org:act:61043D65-F634-4D8C-9904-01EC2163CC42

http://species-id.net/wiki/Sperchon_orbipatella

**Figures 11–13.**
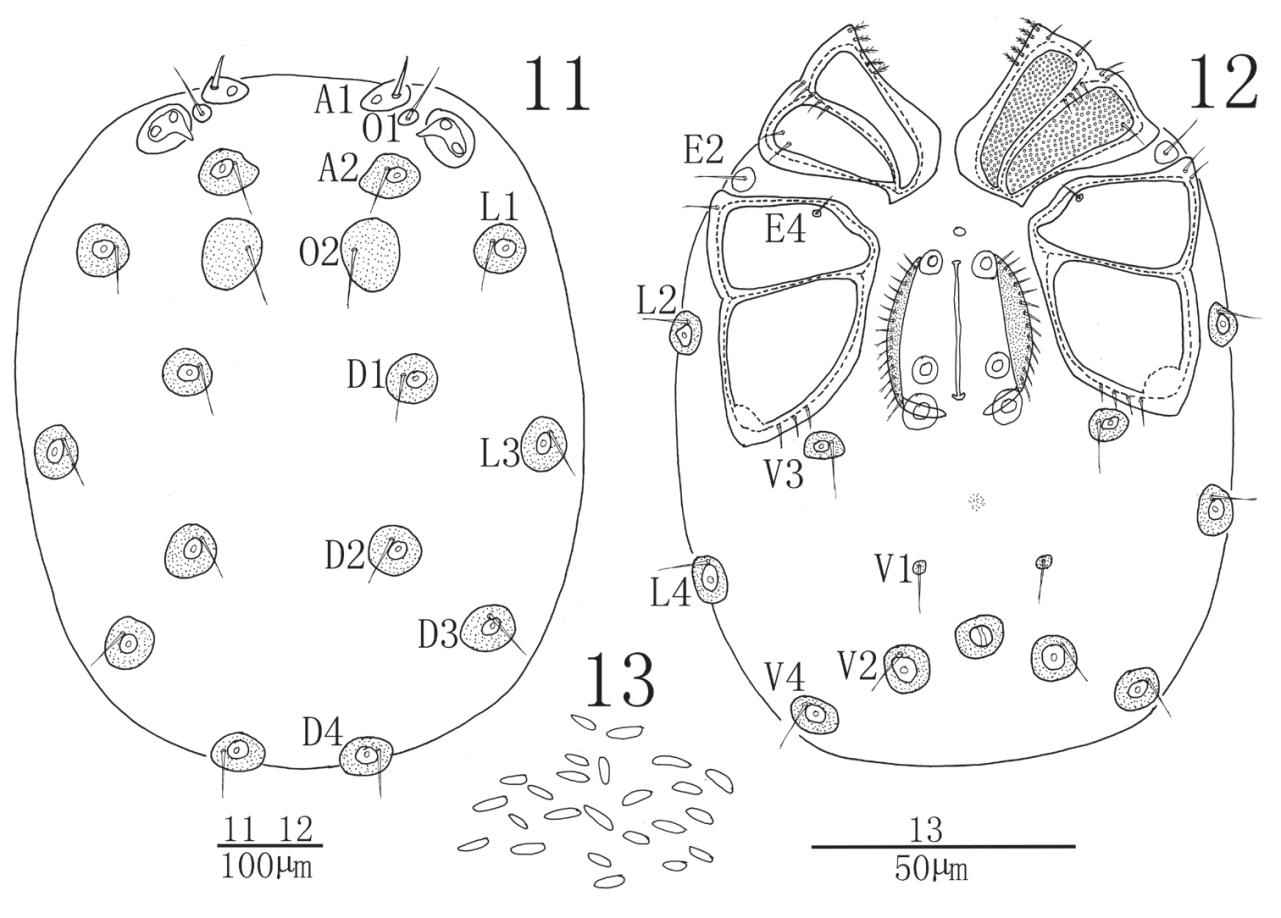
*Sperchon (Sperchon) orbipatella* sp. n., Male **11** idiosoma, dorsal view 1**2** idiosoma, ventral view 1**3** decorations of cuticle.

Figures 11 [Fig F5] 21 

###### Type series.

Holotype: Male, Xinjiang Uygur Autonomous Region, Urumqi city, Baiyanggou scenic area, an unnamed stream (43°40'53"N, 88°04'59"E), 18 August 1996, coll. Dao-Chao Jin. Paratypes: 46 males and 59 females, the same data as the holotype.

###### Diagnosis.

Cuticle covered with small papillae various in shape; excretory pore surrounded by a sclerotized ring; the second acetabulum near to the third but far away from the first one; P-II with a long ventro-distal projection and one thick seta at the base of the projection; third to fifth segments of leg I-IV with rather short plumose setae in longitudinal rows.

###### Description.

**Male** (n=3):Idiosoma oval in outline, 636 (602-783) in length, 533 (510-563) in width. Cuticle yellow-brown, covered with small, various shaped papillae (Fig. 13). A1 smooth, short and thick, other glandularia thin and longer. Cuticle without an extended dorsal and ventral plate. Glandularia and O2 encircled by small rounded platelets (Fig. 11, Fig. 12). Coxae in four groups, surface of coxae reticulated. ACG 162 (153-176) in length, close to each other but not fused, posterior apodeme weakly developed. E2 laterally between ACG and PCG. PCG 239 (228-257) in length. E4 close to anterior margin of CxIII. Distance between anterior end of ACG and posterior end of PCG 387 (364-412). Genital field between PCG with a small and rounded platelet in front. Genital valves not covering the genital acetabula, 170 (157-184) in length, 140 (132-165) in width. Pre- and postgenital sclerites not developed. Three pairs of small and rounded genital acetabula, the second acetabulum near to the third and far away from the first. V1 on very small sclerites and without accompanying glandularia. Excretory pore surrounded by a sclerotized ring and placed anterior to the line of V2 glands.

Infracapitulum with a relatively short and high rostrum, length 172 (165-184). Chelicera total length 223 (207-246), basal segment length 165 (155-183), claw length 58 (52-63), basal segment/claw length ratio 2.8 (2.8-3.0). Dorsal lengths of the palpal segments: P-I, 32 (25-34); P-II, 99 (80-107); P-III, 121 (113-134); P-IV, 163 (145-173); P-V, 37 (34-45). P-I short and without seta. P-II with a long ventro-distal projection bearing one thin seta and a thick peg-like seta at the base of the projection, the thick seta almost as long as the projection. Six setae on the dorsal side of the P-II, none of them plumose. The venter margin of P-III without setae, two setae on the dorsal side, one of which almost on middle and another one near to the distal end of the segment. P-IV venter with two subequal peg-like setae, well distanced from each other. Six thin setae on the dorsal side of P-IV, four of them on the terminal end of segment. Dorsal lengths of the first leg: I-L-1, 56 (47-69); I-L-2, 71 (58-86); I-L-3, 78 (62-88); I-L-4, 144 (126-163); I-L-5, 145 (131-167); I-L-6, 143 (123-159). Dorsal lengths of the fourth leg: IV-L-1, 85 (76-93); IV-L-2, 107 (96-114); IV-L-3, 115 (108-127); IV-L-4, 238 (221-257); IV-L-5, 215 (197-226); IV-L-6, 205 (196-214). Third to fifth segments of leg I-IV with rather short plumose setae in longitudinal rows (Fig. 19). Claws with well protruded claw-blade and two clawlets, a long dorsal and a shorter ventral one (Fig. 15).

**Female** (n = 3):Similar to male except for the morphology of genital field (Fig. 21). Idiosoma 771 (725-947) in length, 645 (617-768) in width. ACG 199 (182-218) in length, PCG 272 (253-304) in length. Distance between anterior end of ACG and posterior end of PCG 448 (436-469). Genital field 209 (182-234) in length, 174 (163-181) in width. Infracapitulum length 210 (197-242). Chelicera total length 283 (262-307), basal segment length 212 (195-230), claw length 71 (67-77), basal segment/claw length ratio 3.0 (2.9-3.0). Dorsal lengths of the palpal segments: P-I, 37 (32-41); P-II, 122 (113-137); P-III, 155 (140-172); P-IV, 197 (178-217); P-V, 48 (40-54). Dorsal lengths of leg I: I-L-1, 67 (54-73); I-L-2, 100 (87-113); I-L-3, 128 (112-147); I-L-4, 217 (203-246); I-L-5, 197 (184-213); I-L-6, 183 (169-212). Dorsal lengths of leg IV: IV-L-1, 95 (87-106); IV-L-2, 148 (132-167); IV-L-3, 151 (137-173); IV-L-4, 299 (267-315); IV-L-5, 280 (262-301); IV-L-6, 227 (209-246).

**Figures 14–19. F5:**
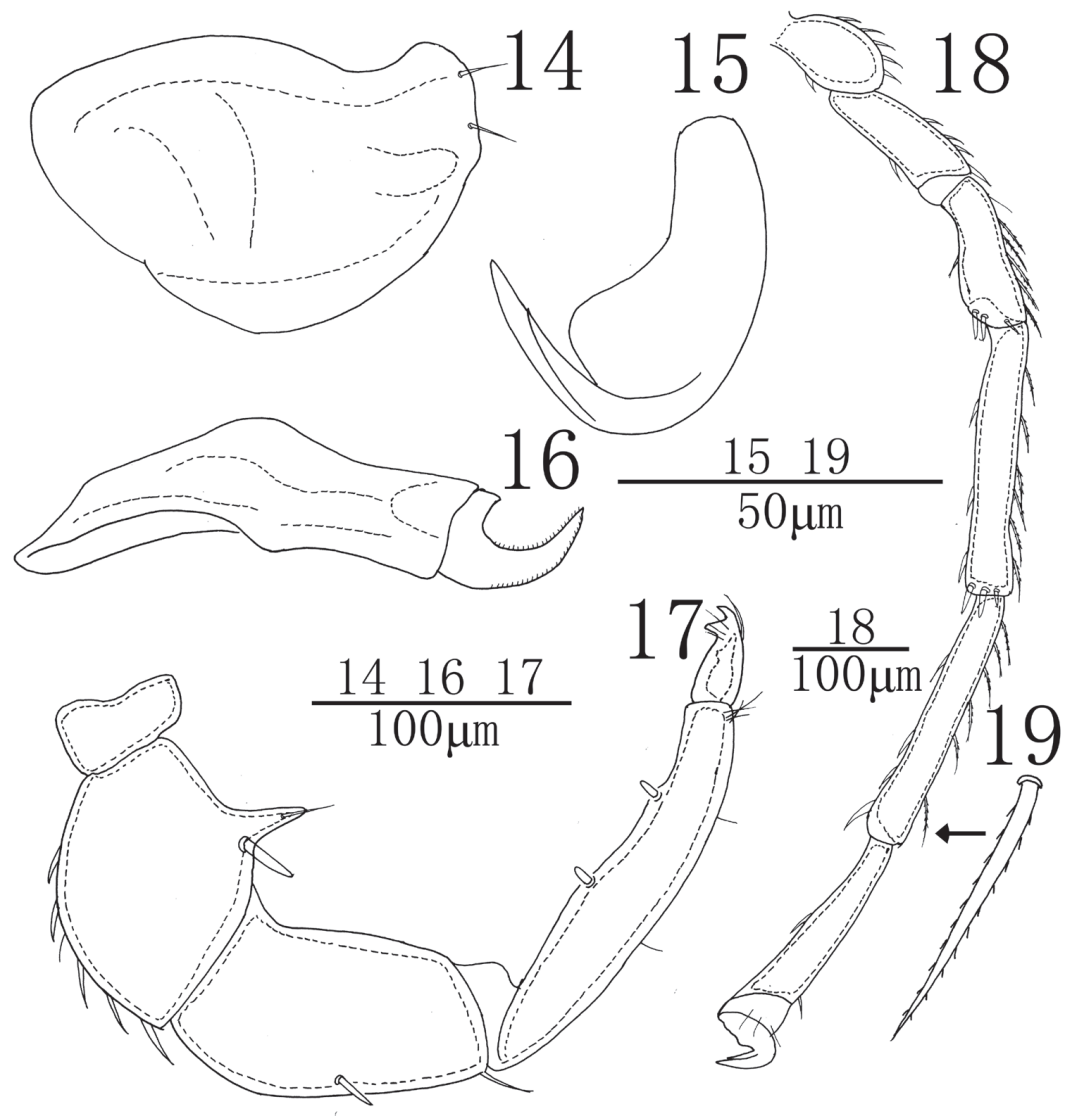
*Sperchon (Sperchon) orbipatella* sp. n., Male 1**4** infracapitulum 1**5** claw 1**6** chelicera **17** palp 1**8** IV-L-1–6 1**9** dorsal seta of IV-L-5.

**Figures 20–21.**
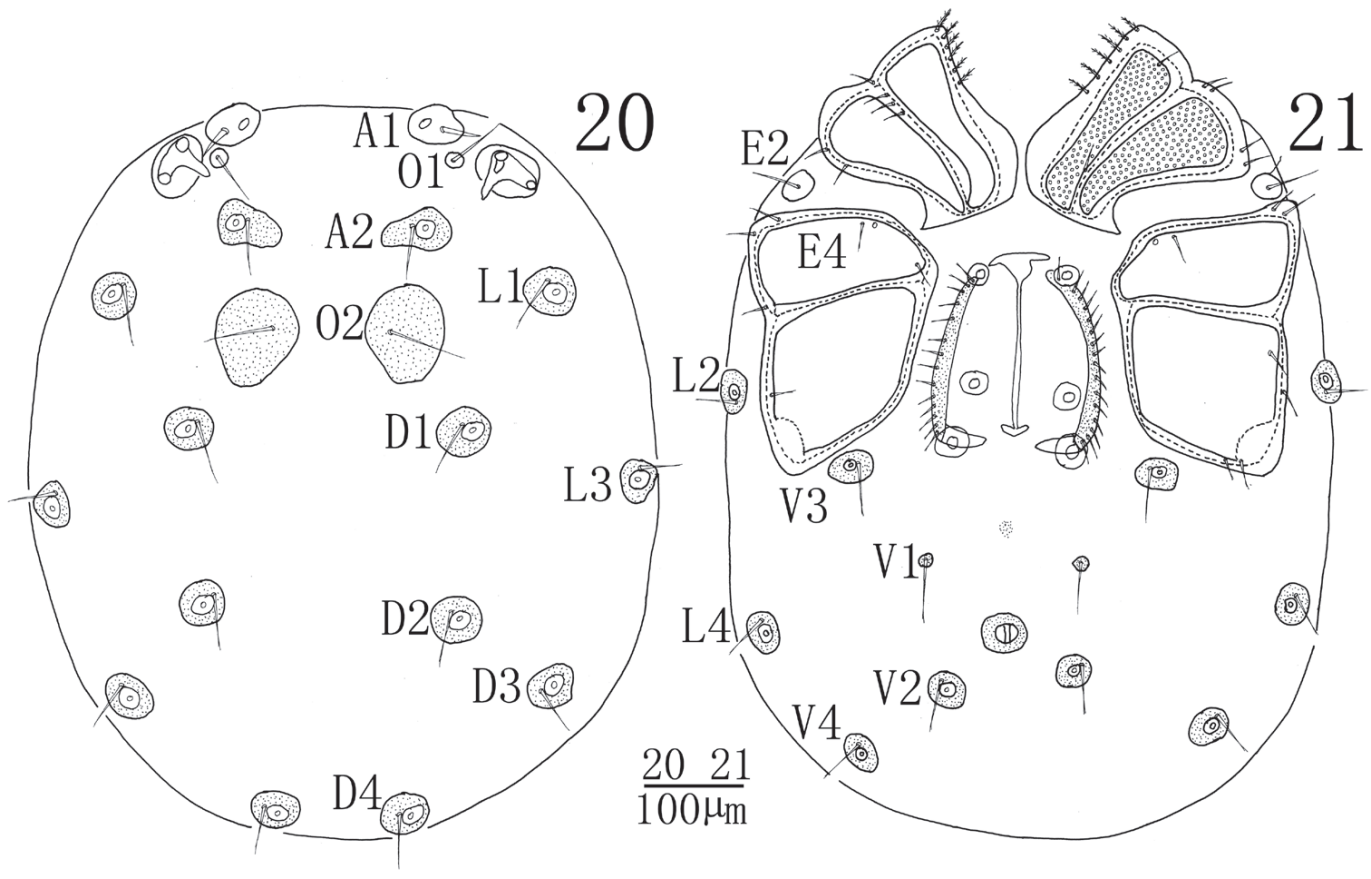
*Sperchon (Sperchon) orbipatella* sp. n., Female **20** idiosoma, dorsal view **21** idiosoma, ventral view.

###### Etymology.

The species is named after the shape of the three acetabula, “orbi-” Latin word, means rounded.

**Remarks.** All the species of the genus *Sperchon* have three pairs of acetabula, in most cases arranged almost equidistantly, usually the anterior two pairs of acetabula elongated while the posterior pair is somewhat rounded. However, in some species such as *Sperchon prosperoides* Tuzovskij, 1990 and *Sperchon minutiporus* Sokolow, 1934, the acetabula are small and rounded, and the second pair shifted near to the third one but far away from the first one.

The new species resembles to *Sperchon prosperoides* Tuzovskij, 1990, from Russia, from which it can been distinguished by the morphology of the palp and genital field (Tuzovskij 2008). In *Sperchon prosperoides*, P-III bearing two thick setae on the ventral side and only half of the genital field is located between PCG, whereas in the new species, P-III is without setae on the ventral side and the genital field is located entirely between PCG.

###### Distribution.

China (Xinjiang Uygur Autonomous Region).

##### Sperchon (Sperchon) sounkyo

Imamura, 1954

http://species-id.net/wiki/Sperchon_sounkyo

**Figures 22–24.**
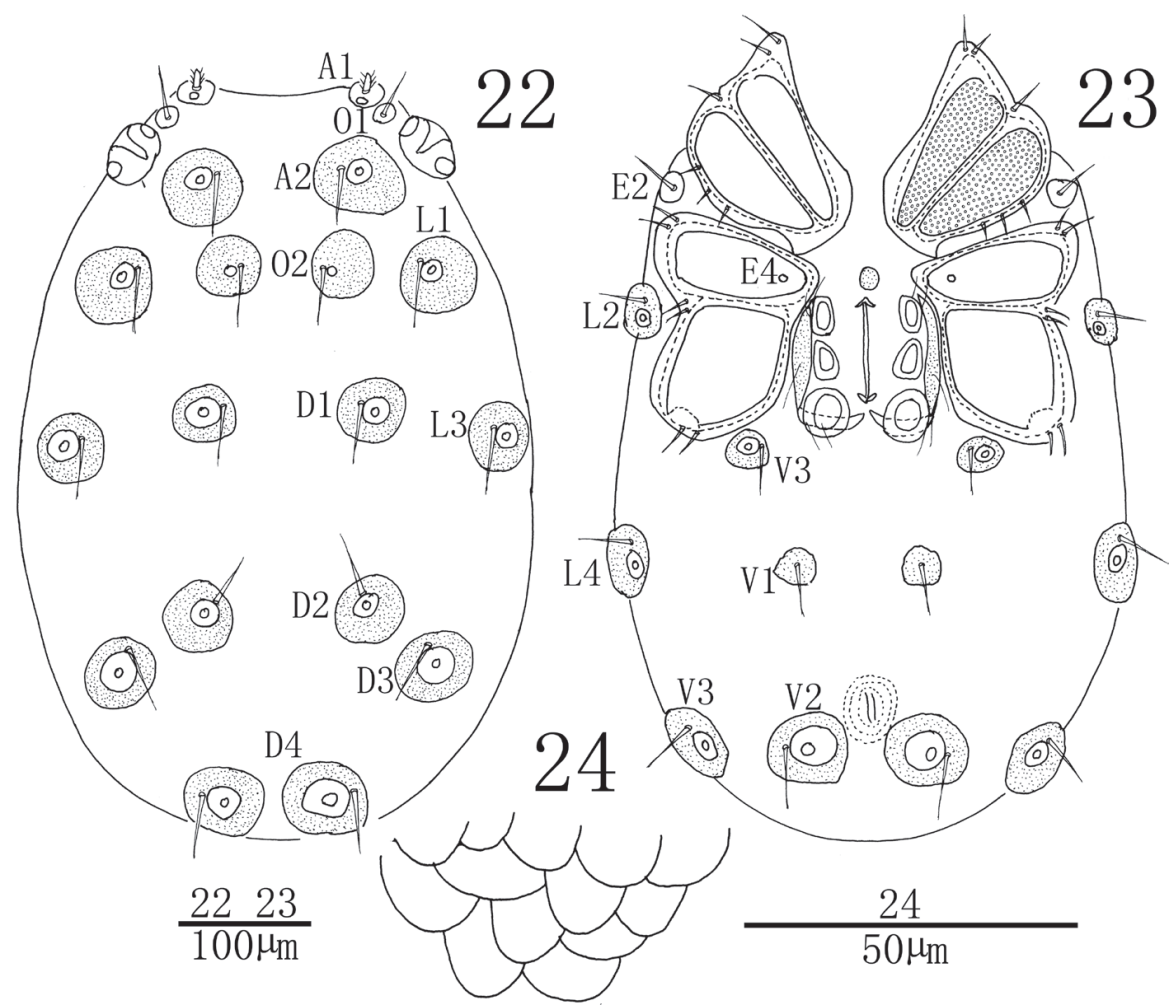
*Sperchon (Sperchon) sounkyo* Imamura, 1954, Male **22** idiosoma, dorsal view **23** idiosoma, ventral view **24** decorations of cuticle.

Figures 22 [Fig F8] 31 

###### Material examined.

25 males and 36 females, Xinjiang Uygur Autonomous Region, Altay City, Kanas Lake (48°48'59"N, 87°02'01"E), 14 August 1997, coll. Dao-Chao Jin; 2 males, Shanxi Province, Taibaishan Mountain National Forest Park, Honghuaping area scene, an unnamed stream (34°07'16"N, 107°53'46"E), 02 September 1997, coll. Dao-Chao Jin; 3 females, Hebei Province, Wulingshan National Nature Reserve, an unnamed stream (40°37'23"N, 117°29'51"E), 03 April 2002, coll. Jian-Jun Guo; 2 males and 1 female, Guizhou Province, Xishui National Natrue Reserve, an unnamed stream (28°28'19"N, 106°05'41"E), 02 Jun 2002, coll. Jian-Jun Guo; 10 males and 8 females, Guizhou Province, Kuankuoshui National Natrue Reserve, an unnamed stream (28°14'25"N, 107°11'59"E), 22 May 2004, coll. Zhen-Zao Tian and Ai-Ping Cui; 11males and 9 females, Guizhou Province, Leigongshan National Nature Reserve, an unnamed stream (26°21'24"N, 104°34'40"E), 21 May 2005, coll. Xu Zhang.

###### Description.

**Male** (n = 4):Idiosoma oval in outline, 561 (523–635) in length, 396 (374-418) in width. Cuticle yellow-brown, covered with scale-shaped papillae (Fig. 24). A1 short and plumose, setae of other glandularia smooth and relatively long. Cuticle without dorsalia and ventralia except the platelets surrounding glandularia and O2 (Fig. 22, Fig. 23). Coxae in four groups, surface of coxae reticulated. ACG 157 (148-166) in length, apodeme developed. E2 laterally between ACG and PCG. PCG 167 (160-174) in length. E4 on the median and near to inner margin of CxIII. Distance between anterior end of ACG and posterior end of PCG 308 (283-317). Genital field between PCG with a small and rounded platelet in front. Genital valves not covering the genital acetabula, 113 (108-121) in length, 109 (101-114) in width. Pre- and postgenital sclerites visible though not developed. Three pairs of genital acetabula, the first pair of genital acetabula more or less rectangular, the second pair somewhat triangular, and the third pair rounded and larger than the anterior two. V1 on median size sclerites and without accompanying glandularia. Excretory pore without sclerotized ring, and anterior to the line of V2 glands.

Infracapitulum with a short rostrum, length 193 (185-203). Chelicera total length 204 (194-219), basal segment length 151 (146-160), claw length 53 (48-59), basal segment/claw length ratio 2.8 (2.7-3.0). Dorsal lengths of the palpal segments: P-I, 21 (19-23); P-II, 94 (85-102); P-III, 102 (94-108); P-IV, 97 (92-111); P-V, 24 (22-27). P-I short and without seta. P-II with a long ventro-distal projection, one thin and long seta near to the base of the projection. Four setae on the dorsal and lateral side of the P-II, one of them plumose. The ventral side of P-III nearly straight and without seta, six short smooth setae on the lateral and dorsal side. P-IV with two small peg-like ventral setae and three thin setae, the proximal peg-like seta is larger than the distal one. The dorsal of P-IV with three thin setae, two of them located distally. Dorsal lengths of the first leg: I-L-1, 44 (39-50); I-L-2, 63 (55-71); I-L-3, 68 (59-76); I-L-4, 119 (112-138); I-L-5, 109 (102-123); I-L-6, 105 (97-114). Dorsal lengths of the fourth leg: IV-L-1, 61 (57-65); IV-L-2, 85 (76-95); IV-L-3, 103 (92-116); IV-L-4, 195 (183-210); IV-L-5, 204 (191-218); IV-L-6, 162 (149-178). Third to fifth segments of leg I-IV with rather short smooth setae in longitudinal rows. Ambulacrum with two claws and each claw with protruded claw-blade and two clawets, a long dorsal and a shorter ventral one (Fig. 28).

**Female** (n = 3):Similar to male except for the morphology of genital field and the size of idiosoma (Fig. 30, Fig. 31). Idiosoma 910 (768-944) in length, 570 (493-648) in width. ACG 182 (170-190) in length, PCG 215 (204-221) in length. Distance between anterior end of ACG and posterior end of PCG 397 (381-405). Genital field 174 (164-181) in length, 154 (144-162) in width. Pre- and postgenital sclerites more developed than in the male. Infracapitulum length 229 (213-238). Chelicera total length 270 (246-279), basal segment length 200 (183-205), claw length 70 (63-74), basal segment/claw length ratio 2.9 (2.7-2.9). Dorsal lengths of the palpal segments: P-I, 23 (20-25); P-II, 123 (118-130); P-III, 149 (142-156); P-IV, 113 (107-122) ; P-V, 29 (26-33). Dorsal lengths of leg I: I-L-1, 43 (37-49); I-L-2, 65 (60-74); I-L-3, 85 (79-97); I-L-4, 147 (136-154); I-L-5, 134 (125-147); I-L-6, 112 (106-120). Dorsal lengths of leg IV: IV-L-1, 75 (70-82); IV-L-2, 111 (103-119); IV-L-3, 118 (112-127); IV-L-4, 242 (221-263); IV-L-5, 223 (208-237); IV-L-6, 187 (178-196).

**Figures 25–29. F8:**
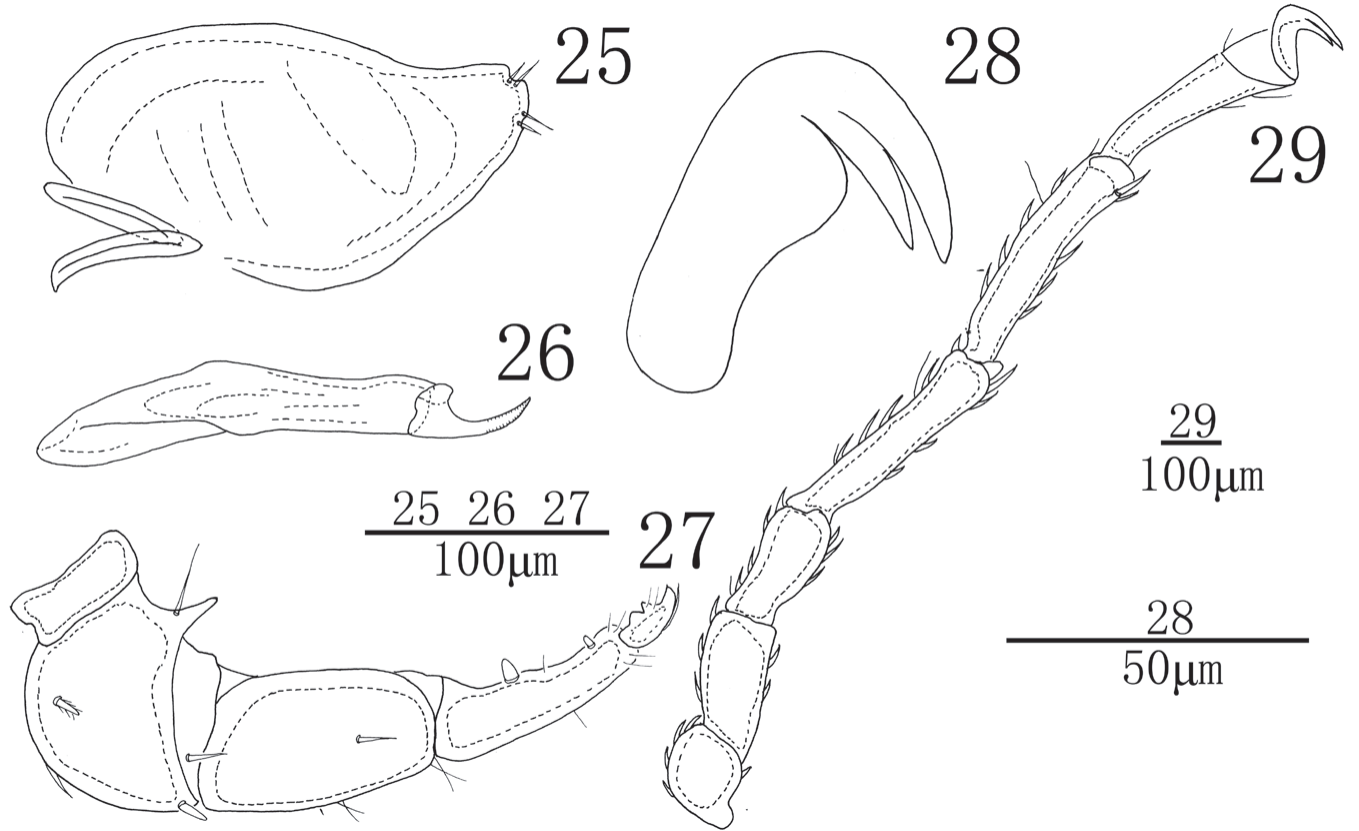
*Sperchon (Sperchon) sounkyo* Imamura, 1954, Male **25** infracapitulum **26** chelicera **27** palp**28** claw **29** IV-L-1–6.

**Figures 30–31.**
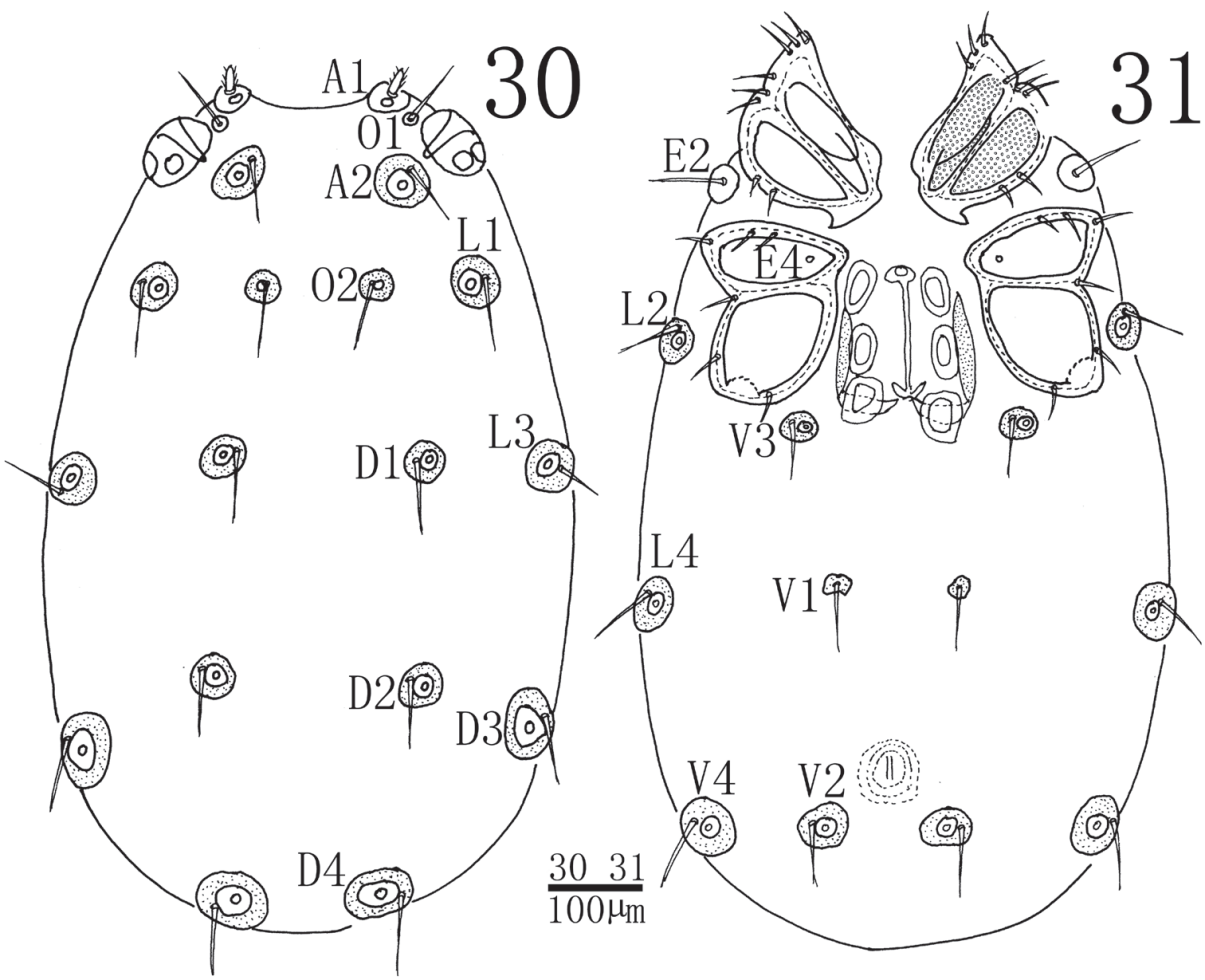
*Sperchon (Sperchon) sounkyo* Imamura, 1954, Female **30** idiosoma, dorsal view **31** idiosoma, ventral view.

###### Remarks.

*Sperchon sounkyo* Imamura, 1954, was firstly described from Japan based on a single female (Imamura 1954), and never recorded since the first description.

Although the original descriptions and illustrations of *Sperchon sounkyo* were lacking of some specific features, such as shape of A1 (Fig. 22, Fig. 30) and leg claws (Fig. 28), most characters of our specimens (e.g., shape of cuticle and palp, E4 on CxIII, and excretory pore without sclerotized ring) are in accordance with this species. So we attribute our specimens to *Sperchon sounkyo* Imamura, 1954. Some differences found in the body size and the number of the setae on the palpal between the Chinese specimens and Japanese specimens should be regarded as the variety between the different populations.

It is the first report from China and the first description of the male of *Sperchon sounkyo*. This species seems to be a widely distributed species in China.

###### Distribution.

China (present study); Japan (Imamura 1954).

##### Sperchon (Sperchon) urumqiensis sp. n.

urn:lsid:zoobank.org:act:53DAC024-9964-47BA-BA72-B3563069B059

http://species-id.net/wiki/Sperchon_urumqiensis

**Figures 32–34.**
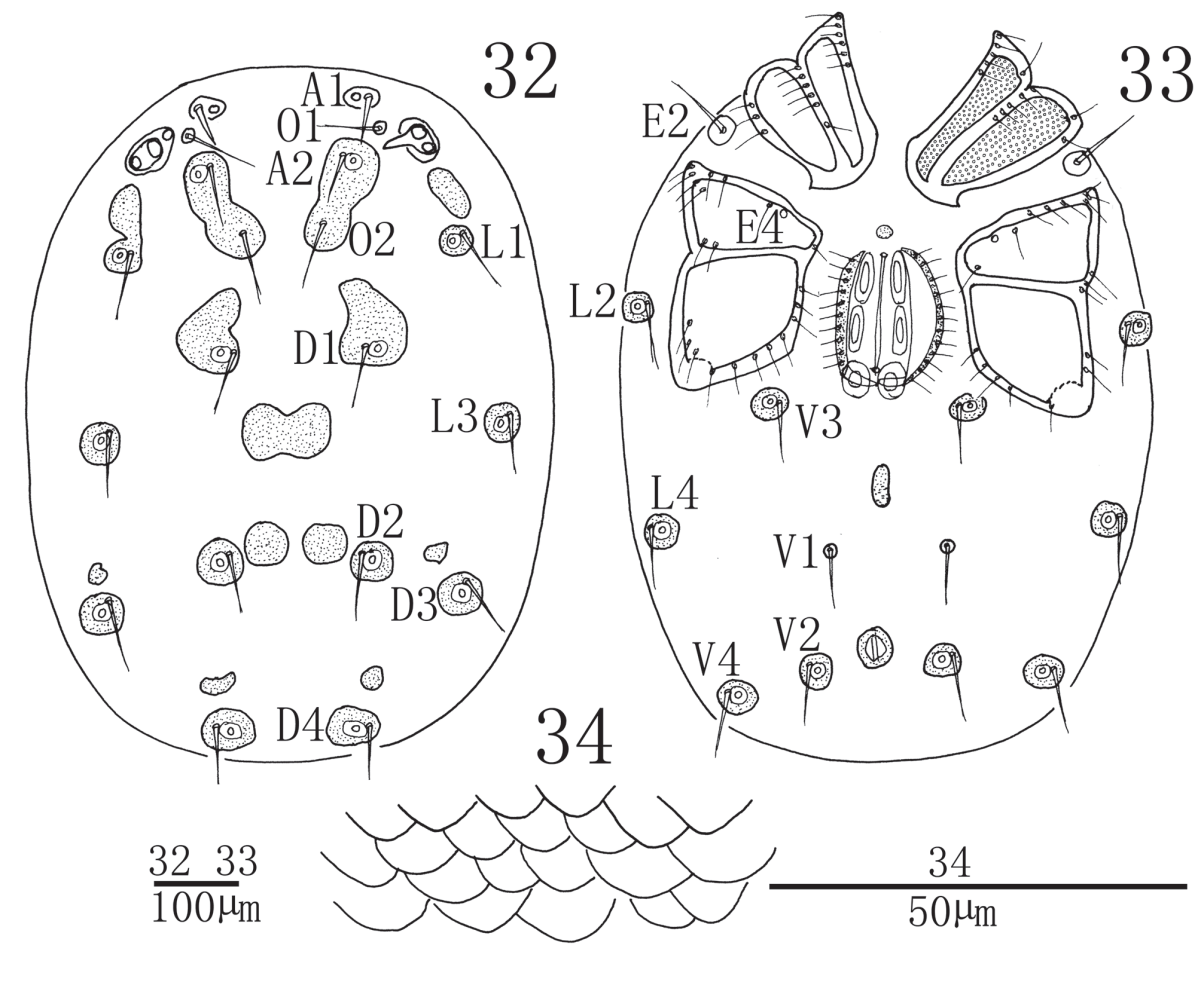
*Sperchon (Sperchon) urumqiensis* sp. n., Male **32** idiosoma, dorsal view **33** idiosoma, ventral view **34** decorations of cuticle.

Figures 32 [Fig F11] 42 

###### Type series.

Holotype: Male, Xinjiang Uygur Autonomous Region, Urumqi city, Baiyanggou scenic area, an unnamed stream (43°37'42"N, 87°57'18"E), 18 Aug 1996, coll. Dao- Chao Jin. Paratypes: 6 males and 8 females, the same data as the holotype.

###### Diagnosis.

Cuticle covered with scale-shaped papillae; excretory pore surrounded by a sclerotized ring; P-II with a long ventro-distal projection and one heavy seta close to the ventrodistal margin of P-II; third to fifth segments of leg I-IV with rather long plumose setae in longitudinal rows.

###### Description.

**Male** (n = 3):Idiosoma oval in shape, 812 (756-907) in length, 682 (663-712) in width. Cuticle yellow in colour, covered with scale-shaped papillae (Fig. 34). A1 smooth, short and thick, other glandularia thin and long. All glandularia and O2 encircled by a platelet. Some chitinous platelets and glandular platelets on dorsum fused as shown in Fig. 32. Coxae in four groups, surface of coxae reticulated. ACG 163 (158-171) in length, posterior apodeme weakly developed. E2 laterally between ACG and PCG. PCG 242 (236-254) in length. E4 close to anterior margin of CxIII. Distance between anterior end of ACG and posterior end of PCG 443 (432-464). Genital field between PCG with a small and rounded platelet in front. Genital valves not covering the genital acetabula, 188 (182-193) in length, 117 (113-124) in width. Pre- and postgenital sclerites not developed. Three pairs of acetabula, the two anterior pairs of acetabula long elliptic and the posterior pair more or less rounded. An oblong plate posterior to genital field, almost in the middle line of V1 and V3. V1 on very small sclerites and without accompanying glandularia. Excretory pore surrounded by a sclerotized ring and close to the line at V2 glands.

Infracapitulum with a short and heavy rostrum, length 189 (185-197). Chelicera total length 229 (225-237), basal segment length 174 (172-179), claw length 55 (53-58), basal segment/claw length ratio 3.2 (3.1–3.2). Dorsal lengths of the palpal segments: P-I, 22 (21-24); P-II, 99 (97-106); P-III, 118 (113-127); P-IV, 171 (165-182); P-V, 37 (34-42). P-I short and with one dorsal seta. P-II with a long ventro-distal projection bearing two thin setae, of which one longer than another. One heavy seta close to the ventrodistal margin of P-II. Seven setae on the dorsal and lateral side of the P-II, none of them plumose. The ventral side of P-III somewhat swelled and without seta, two long smooth setae on the lateral and dorsal side. P-IV slender, much longer than P-III, and with two ventral peg-like setae, of which the proximal one almost at ventral middle of the segment, and the distal one approximately equidistant from proximal one and distal end of the segment. Four thin setae on P- IV, one of them on the median dorsal, two on the dorsal distal and another one on the terminal end of the segment. Dorsal lengths of the first leg: I-L-1, 39 (36-43); I-L-2, 83 (80-90); I-L-3, 98 (90-106); I-L-4, 191 (185-200); I-L-5, 182 (177-193); I-L-6, 179 (174-187). Dorsal lengths of the fourth leg: IV-L-1, 87 (85-94); IV-L-2, 117 (111-128); IV-L-3, 154 (148-168); IV-L-4, 300 (290-317); IV-L-5, 271 (264-285); IV-L-6, 257 (249-266). Third to fifth segments of leg I-IV with rather long plumose setae in longitudinal rows (Fig. 39). Ambulacrum with two claws and each claw with well protruded claw-blade and two clawets, a long dorsal and a shorter ventral one (Fig. 37).

**Female** (n = 3):Color, idiosoma shape, and the decorations of cuticle as in the male. Characters of the genital field, the fusion and the size of dorsalia and ventralia different from the male (Fig. 41, Fig. 42). Idiosoma 979 (953-1023) in length, 829 (805-876) in width. ACG 202 (197-212) in length, PCG 301 (295-314) in length. Distance between anterior end of ACG and posterior end of PCG 559 (547-578). Genital field 227 (223-234) in length, 245 (241-253) in width. Pre- and postgenital sclerites well developed than that in the male. Infracapitulum length 255 (250-261). Chelicera total length 317 (311-331), basal segment length 244 (241-253), claw length 73 (70-78), basal segment/claw length ratio 3.3 (3.2-3.4). Dorsal lengths of the palpal segments: P-I, 47 (45-50); P-II, 146 (140-155); P-III, 181 (174-193); P-IV, 274 (263-289); P-V, 45 (42-49). Dorsal lengths of leg I: I-L-1, 70 (64-78); I-L-2, 135 (127-148); I-L-3, 128 (122-137); I-L-4, 256 (243-171); I-L-5, 272 (260-284); I-L-6, 234 (222-247). Dorsal lengths of leg IV: IV-L-1, 115 (119-124); IV-L-2, 156 (148-169); IV-L-3, 230 (219-244); IV-L-4, 379 (366-397); IV-L-5, 363 (351-380); IV-L-6, 322 (311-340).

**Figures 35–40. F11:**
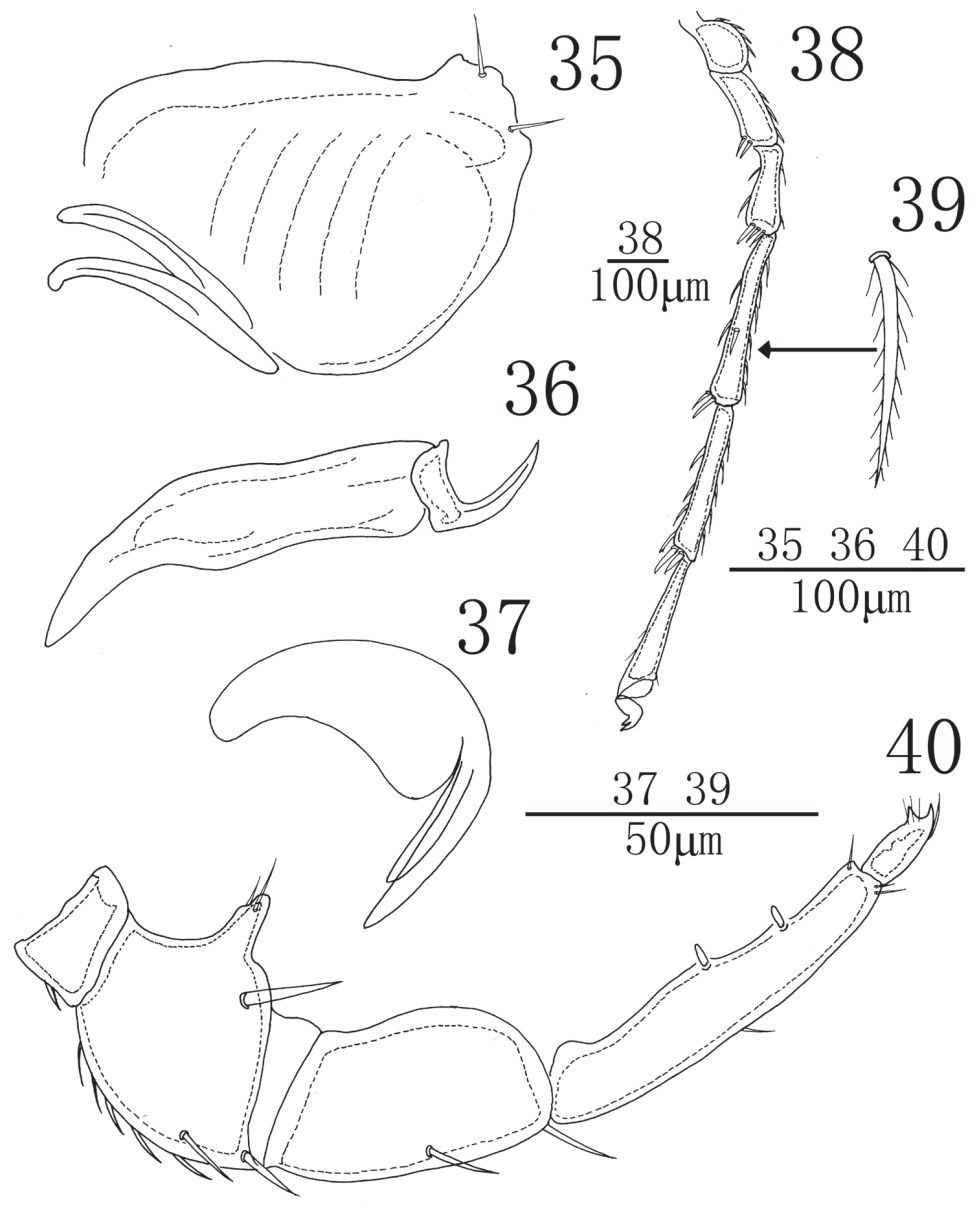
*Sperchon (Sperchon) urumqiensis* sp. n., Male **35** infracapitulum; **36** chelicera **37** claw palp**38** IV-L-1–6 **39** dorsal seta of IV-L-4 **40** palp.

**Figures 41–42.**
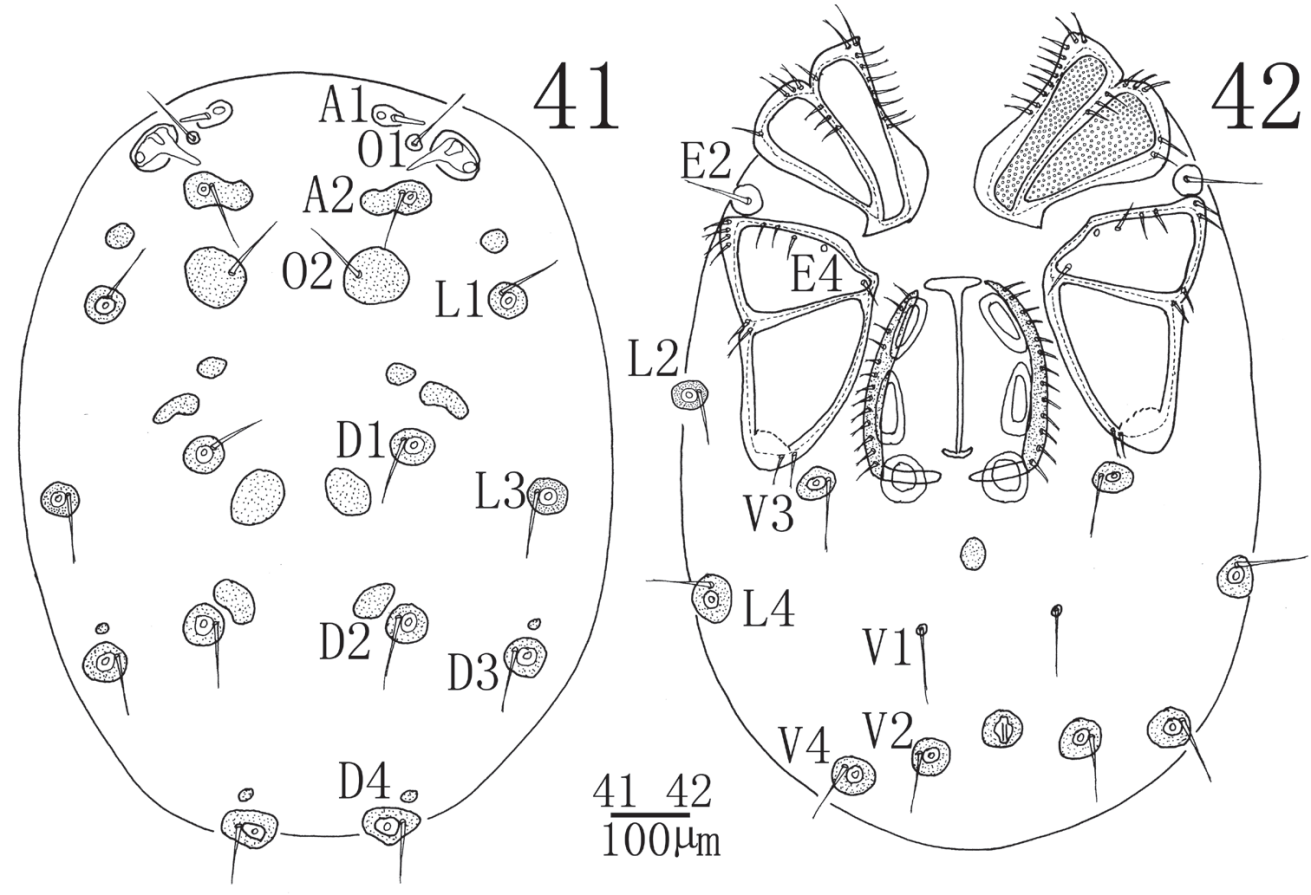
*Sperchon (Sperchon) urumqiensis* sp. n., Female **41** idiosoma, dorsal view **42** idiosoma, ventral view.

###### Etymology.

The species is named after the city where it was collected.

###### Remarks.

Due to the shape of cuticle, E4 on the CxIII and P-II with a long ventro-distal projection, the new species is similar to *Sperchon sounkyo* Imamura, 1954. From the later species, *Sperchon urumqiensis* sp. n. can be easily distinguished by the following features: the dorsum and posterior venter with sclerotized muscle attachment plates (Fig. 32-33, Fig. 41-42) (unsclerotized in *Sperchon sounkyo*, see Fig. 22-23, Fig. 30-31), the presence of a long and heavy seta close to the ventrodistal margin of P-II (Fig. 40) (vs. without heavy seta in *Sperchon sounkyo*, see Fig. 27), the two peg-like setae of P-IV are subequal in size (Fig. 40) (proximoventral seta larger than distoventral in *Sperchon sounkyo*, see Fig. 27), and the excretory pore is sclerotized (Fig. 33, Fig. 42) (smooth in *Sperchon sounkyo*, see Fig. 23, Fig. 31).

The new species also resemble *Sperchon ussuriensis* Tuzovskij, 2002 from Russia (Tuzovskij 2002). However, in *Sperchon ussuriensis*, the anterior groups of coxae are fused and the excretory pore is unsclerotized. While in the new species, the anterior groups of coxae are not fused and the excretory pore is surrounded by a sclerotized ring.

###### Distribution.

China (Xinjiang Uygur Autonomous Region).

### Genus *Sperchonopsis* Piersig, 1896

#### Subgenus *Sperchonopsella* Conroy, 1991

##### Sperchonopsis (Sperchonopsella) whiteshellensis

Conroy, 1991

http://species-id.net/wiki/Sperchonopsis_whiteshellensis

Figures 43 51 

###### Material examined.

3 males and 6 females, Xinjiang Uygur Autonomous Region, Altay City, Kanas Lake (48°48'52"N, 86°57'47"E), 14 August 1997, coll. Dao-Chao Jin; 24 males and 33 females, Yunnan Province, Tengchong country, Gaoligongshan National Nature Reserve, an unnamed stream (25°33'12"N, 98°35'18"E), 15 July 2002, coll. Jian-Jun Guo.

###### Description.

**Figures 43–49.**
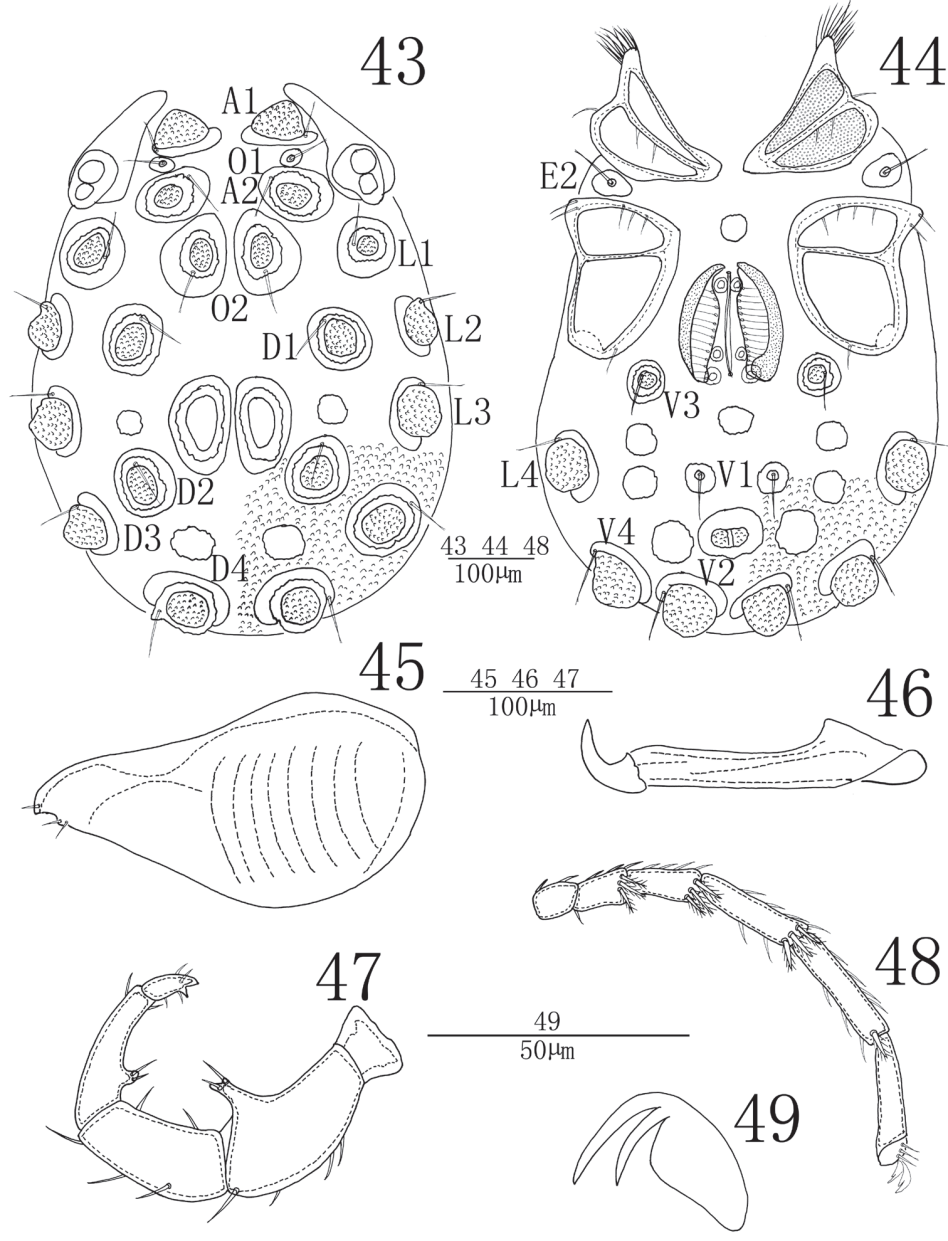
*Sperchonopsis (Sperchonopsella) whiteshellensis* Conroy, 1991,Male **43** idiosoma, dorsal view **44** idiosoma, ventral view **45** infracapitulum **46** chelicera **47** palp **48** IV-L-1–6 **49** claw.

**Male** (n = 4):Idiosoma oval in outline, 578 (562-675) in length, 418 (410-457) in width. Cuticle yellow-brown in color, and covered with papillae. The eye capsules in some young specimens well developed and somewhat projected over the idiosoma margin (Fig. 43). Glandularia greatly enlarged and projected with well-developed papillae, and encircled by platelet. Chitinous platelets and glandularia on dorsum and ventrum as showed in Fig. 43 and Fig. 44. Coxae in four groups, surface of coxae reticulated. ACG 152 (151-157) in length, posterior apodeme indistinct. Anterior tip of CxI with a tuft of hair-like setae. E2 laterally between ACG and PCG. PCG 167 (164-175) in length. Glandularia absent from CxIII. Approximately two-thirds of genital field lying between PCG. Genital valves not covering the genital acetabula, 176 (174-188) in length, 145 (143-152) in width. One round platelet in front of genital field. Pre- and postgenital sclerites small. Three pairs of small and rounded genital acetabula, the second pair much close to the third one and far away from the first one. V1 on sclerites in medium size and without accompanying glandularia. Excretory pore lying between V1 and V2 and on a protuberance surrounded by a well-developed sclerotized ring.

Infracapitulum with a relatively long rostrum, length 196 (194-207). Chelicera total length 207 (204-222), claw length 151 (149-162), basal segment length 56 (55-60), basal segment/claw length ratio 2.7 (2.7). Dorsal lengths of palpal segments: P-I, 24 (24-27); P-II, 104 (101-112); P-III, 94 (92-100); P-IV, 96 (93-105); P-V, 33 (32-36). P-I short and without seta. P-II with a long ventro-distal projection bearing one long and one short setae. About four setae on the lateral and dorsal side of P-II and none of them plumose. P-III with five smooth stae, of them two on the venter and other three on the latero-dorsal side. P-IV without peg-like setae, but with a ventral projection bearing two setae. Two setae on the dorsal and the ventral distal of P-IV respectively. Dorsal lengths of the first leg: I-L-1, 44 (42-49); I-L-2, 43 (42-48); I-L-3, 57 (54-64); I-L-4, 97 (93-108); I-L-5, 87 (84-97); I-L-6, 51 (49-57). Dorsal lengths of the fourth leg: IV-L-1, 70 (67-77); IV-L-2, 68 (62-74); IV-L-3, 88 (85-97); IV-L-4, 168 (160-181); IV-L-5, 169 (158-184); IV-L-6, 152 (148-164). The distal edge of I-IV-L-2–I-IV-L-5 with several thick and plumose setae (Fig. 48). Ambulacrum with two claws and each claw with well protruded claw-blade and two clawets, a long dorsal and a shorter ventral one (Fig. 49).

**Female** (n = 3): Similar to male except the characteristics of genital field and the size of idiosoma. Idiosoma length 886 (853-936), width 626 (607-645). ACG 200 (194-205) in length, PCG 212 (204-221) in length. Distance between anterior end of ACG and posterior end of PCG 438 (424-452). Genital field 213 (207-217) in length, 170 (167-175) in width. Pregenital sclerite well developed. Infracapitulum length 266 (257-276). Chelicera total length 279 (272-289), basal segment length 193 (189-199), claw length 86 (83-90), basal segment/claw length ratio 2.2 (2.2-2.3). Dorsal lengths of the palpal segments: P-I, 36 (34-40); P-II, 155 (148-162); P-III, 132 (126-140); P-IV, 136 (125-143); P-V, 45 (42-48). Dorsal lengths of the first leg: I-L-1, 73 (66-81); I-L-2, 75 (67-83); I-L-3, 84 (77-93); I-L-4, 122 (114-131); I-L-5, 127 (119-138); I-L-6, 108 (101-117). Dorsal lengths of the fourth leg: IV-L-1, 108 (102-117); IV-L-2, 104 (98-112); IV-L-3, 126 (118-135); IV-L-4, 222 (213-233); IV-L-5, 195 (186-207); IV-L-6, 181 (173-190).

**Figures 50–51.**
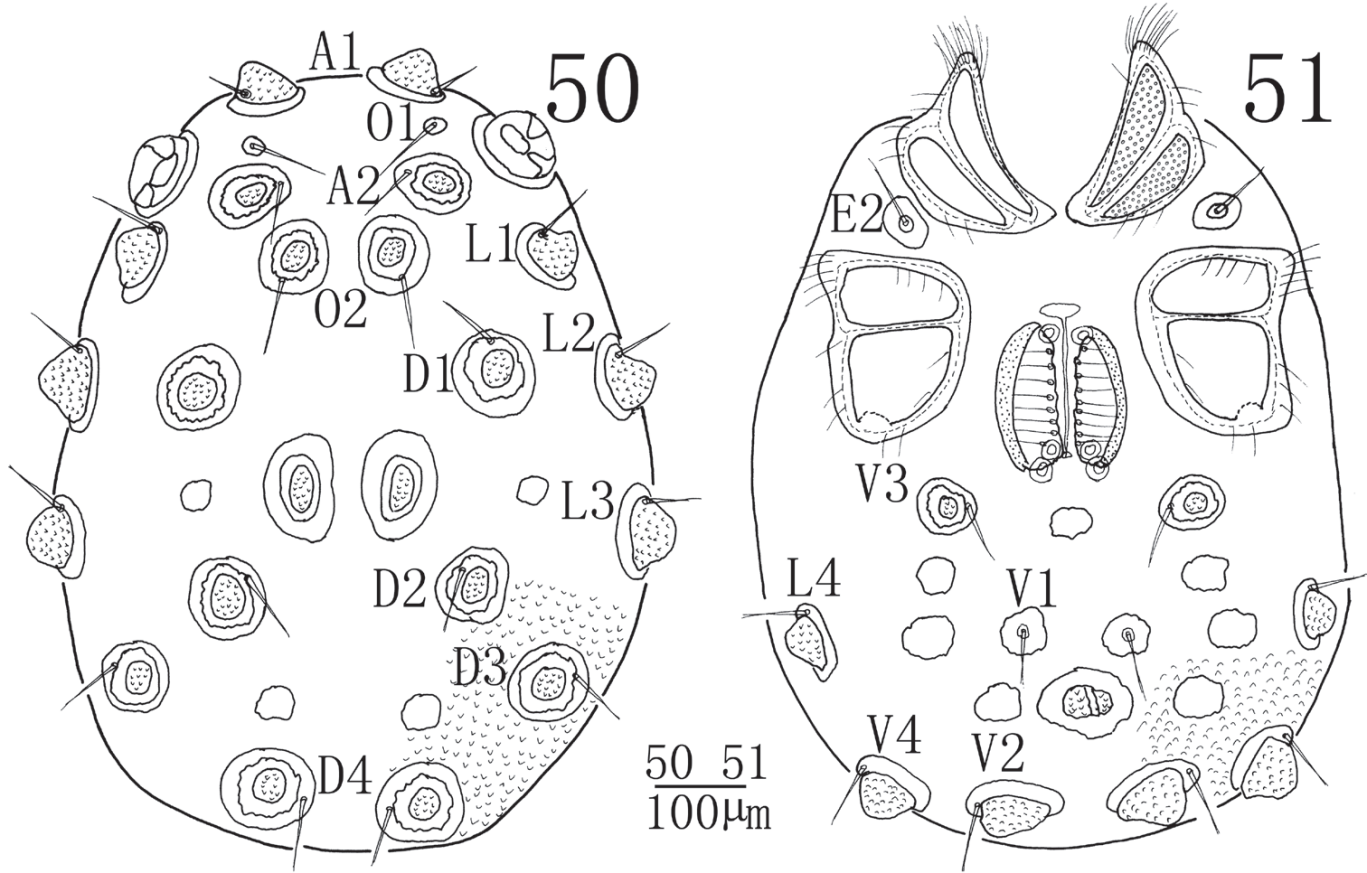
*Sperchonopsis (Sperchonopsella) whiteshellensis* Conroy, 1991, Female **50** idiosoma, dorsal view **51** idiosoma, ventral view.

###### Remarks.

The subgenus *Sperchonopsella* is a small group with only two known species, *Sperchonopsis whiteshellensis* Conroy, 1991 and *Sperchonopsis nipponicus* Uchida, 1934 (Conroy 1991).

Due to the papillate cuticle with the greatly enlarged glandularia, the second and the third pair of acetabula approached to each other and the ventral projections on P-I and P-IV, the specimens from China are similar to *Sperchonopsis whiteshellensis* from North America (Conroy 1991) and *Sperchonopsis nipponicus* from Japan (Uchida 1934). The arrangement of dorsalia and ventralia are in a good agreement with description of *Sperchonopsis whiteshellensis*, and differs from *Sperchonopsis nipponicus* (see illustration in Uchida 1934). Hence we assigned our specimens to *Sperchonopsis whiteshellensis*. This is the first record of the subgenus *Sperchonopsella* from China.

###### Distribution.

China (present study); North America (Conroy 1991)

## Supplementary Material

XML Treatment for Sperchon (Palpisperchon) nikkoensis

XML Treatment for Sperchon (Sperchon) orbipatella

XML Treatment for Sperchon (Sperchon) sounkyo

XML Treatment for Sperchon (Sperchon) urumqiensis

XML Treatment for Sperchonopsis (Sperchonopsella) whiteshellensis

## References

[B1] Conroy JC (1991) New species in the genus *Sperchonopsis* in North America with a description of a new subgenus, *Sperchonopsella*. II. Acarologia 32: 151-161

[B2] Cook DR (1974) Water mite genera and subgenera. Memoirs of the American Entomological Institute 21: 1-860

[B3] Di Sabatino A Gerecke R Gledhill T Smit H (2010) Chelicerata: Acari II. In: Gerecke R (Ed) 2006. Chelicerata: Araraneae, Acari I. *Süßwasserfauna von Mitteleuropa,* Vol. 7, 2–2, Elsevier Spektrum Akademischer Verlag, München, 134 pp.

[B4] Di Sabatino A Smit H Gerecke R Goldschmidt T Matsumoto N Cicolani B (2008) Global diversity of water mites (Acari, Hydrachnidia; Arachnida) in freshwater. Hydrobiologia 595: 303-315 doi: 10.1007/s10750-007-9025-1

[B5] Imamura T (1954) Studies on water-mites from Hokkaido. Journal of Hokkaido Gakugei University, Section B, Supplement 1: 1-148

[B6] Imamura T (1976) Two New Species of Water-Mites from Nikko National Park, Japan. Annotationes zoologicae Japonenses 49 (4): 279-284

[B7] Jin DC (1997)Hydrachnellae: morphology, systematics; a primary study of Chinese fauna. Guizhou science and technology publishing house, Guiyang, 356 pp.[in Chinese]

[B8] Jin DC Yi TC Guo JJ (2010) A review of progress in taxonomy of water mites from China (Acari: Hydrachnidia). Zoosymposia 4: 106-119

[B9] Tuzovskij PV (2002) A new species of the water mite genus *Sperchon* Kramer from Primorsk territory of Russia (Acariformes: Sperchonidae). Zoosystematica Rossica 11 (1): 105-108

[B10] Tuzovskij PV (2008) The morphology of three species of adult water mites of the genus *Sperchon* (*S. kuluensis*, *S. prosperoides*, and *S. orientalis*, Acariformes, Sperchontidae). Entomological Review 88 (2): 139-149 doi: 10.1134/S0013873808020024

[B11] Uchida T (1934) Some rheophilous water-mites from Japan. Journal of the Faculty of Science, Hokkaido Imperial University, Series VI (Zoology) 3 (2): 67-116

[B12] Viets KO (1987) Die Milben des Süßwassers (Hydrachnellae und Halacaridae [part.], Acari). 2. Katalog. Sonderbände des Naturwiss. Vereins Hamburg 8: 1-1012

[B13] Zhang X Jin DC (2010) Three new species and one new record of the subgenus *Hispidosperchon* Thor, 1901 within the genus *Sperchon* Kramer, 1877 from China. Zootaxa 2684: 14-24

[B14] Zhang X Jin DC Guo JJ (2011) Two new water mites species of the genus *Sperchon* Kramer, 1877 (Acari, Hydrachnidia, Sperchontidae). Acta Zootaxonomica Sinica 36 (2): 221-226

[B15] Zhang X Jin DC Guo JJ Yi TC (2010) Descriptions of two new species of *Mixosperchon* Viets, a newly recorded subgenus of water mites, from China (Acari, Hydrachnellae, Sperchontidae). Acta Zootaxonomica Sinica 35 (3): 435-439

[B16] Zhang X Jin DC Guo J J Zhu Q (2007) Three species of the Family Sperchontidae (Acari, Hydrachnellae, Sperchontidae) as new records from China. Sciencepaper Online. (2007–12–28):1–5. [in Chinese] http://www.paper.edu.cn/index.php/default/releasepaper/downPaper/200712-845/2

